# “Every Time I Go in There, It Gives Me Time to Reflect”: A Qualitative Study of Patient Perspectives on Substance Use, Medications for Opioid Use Disorder, and Harm Reduction Following Hospitalization for Serious Injection-Related Infection

**DOI:** 10.1093/ofid/ofaf201

**Published:** 2025-04-03

**Authors:** Thisara Jayasinghe, Mari-Lynn Drainoni, Alexander Walley, Christine Grella, Adam Majeski, Andrew Rolles, Ally Cogan, Guhan Venkatesan, Michael D Stein, Marc Larochelle, Jeffrey H Samet, Simeon D Kimmel

**Affiliations:** Section of General Internal Medicine, Department of Medicine, Boston University Chobanian & Avedisian School of Medicine and Boston Medical Center, Boston, Massachusetts, USA; Section of Infectious Diseases, Department of Medicine, Boston University Chobanian & Avedisian School of Medicine and Boston Medical Center, Boston, Massachusetts, USA; Department of Health, Law and Policy, Boston University School of Public Health, Boston, Massachusetts, USA; Evans Center for Implementation and Improvement Sciences, Boston University, Boston, Massachusetts, USA; Section of General Internal Medicine, Department of Medicine, Boston University Chobanian & Avedisian School of Medicine and Boston Medical Center, Boston, Massachusetts, USA; Semel Institute of Neuroscience and Human Behavior, David Geffen School of Medicine, University of California-Los Angeles, California, USA, and the Lighthouse Institute, Chestnut Health Systems, Chicago, Illinois, USA; Section of General Internal Medicine, Department of Medicine, Boston University Chobanian & Avedisian School of Medicine and Boston Medical Center, Boston, Massachusetts, USA; Section of General Internal Medicine, Department of Medicine, Boston University Chobanian & Avedisian School of Medicine and Boston Medical Center, Boston, Massachusetts, USA; Section of General Internal Medicine, Department of Medicine, Boston University Chobanian & Avedisian School of Medicine and Boston Medical Center, Boston, Massachusetts, USA; Section of General Internal Medicine, Department of Medicine, Boston University Chobanian & Avedisian School of Medicine and Boston Medical Center, Boston, Massachusetts, USA; Department of Health, Law and Policy, Boston University School of Public Health, Boston, Massachusetts, USA; Section of General Internal Medicine, Department of Medicine, Boston University Chobanian & Avedisian School of Medicine and Boston Medical Center, Boston, Massachusetts, USA; Section of General Internal Medicine, Department of Medicine, Boston University Chobanian & Avedisian School of Medicine and Boston Medical Center, Boston, Massachusetts, USA; Department of Community Health Sciences, Boston University School of Public Health, Boston, Massachusetts, USA; Section of General Internal Medicine, Department of Medicine, Boston University Chobanian & Avedisian School of Medicine and Boston Medical Center, Boston, Massachusetts, USA; Section of Infectious Diseases, Department of Medicine, Boston University Chobanian & Avedisian School of Medicine and Boston Medical Center, Boston, Massachusetts, USA

**Keywords:** harm reduction, medications for opioid use disorder, people who inject drugs, serious injection-related infection, substance use disorder treatment

## Abstract

**Background:**

Serious injection-related infections (SIRIs) have high morbidity and mortality, in part from incomplete antibiotic treatment, ongoing substance use and reinfection. Understanding how hospitalizations for SIRIs affect patient perspectives on substance use, harm reduction, and medications for opioid use disorder (MOUD) in the era of hospital-based addiction services will inform efforts to improve care.

**Methods:**

We conducted qualitative interviews at Boston Medical Center with individuals hospitalized with SIRIs between 2020 and 2024. To ensure diverse experiences, we recruited qualifying participants based on record of SIRI International Classification of Diseases, 10th Revision, codes, presence on the outpatient parenteral antibiotic program list, during hospitalizations, and from a drop-in harm reduction program. Interviews were transcribed, coded inductively, and analyzed for key themes.

**Results:**

Participants with SIRIs (n = 30) had the following characteristics: Most had endocarditis (n = 10) or osteomyelitis (n = 9) and had completed the recommended antibiotics (n = 24); the mean age was 39; most were male (n = 19), White (n = 21), and housed (n = 18). Three key themes emerged after SIRI hospitalization: (1) reduced substance use and adoption of harm reduction practices were common; (2) perspectives on MOUD varied, but negative experiences and medication stigma persisted; and (3) SIRI hospitalizations were viewed as an opportunity for reflection on substance use and health.

**Conclusions:**

SIRI hospitalizations and the postdischarge period are opportunities to engage patients in addiction and infectious disease care. Participants expressed ambivalence about MOUD despite access to robust hospital-based addiction medicine services. Longitudinal support that explicitly includes harm reduction and MOUD, both linkage and retention, is needed to improve care for people with SIRIs.

Serious injection-related infections (SIRIs) such as endocarditis, bloodstream infection, septic arthritis, osteomyelitis, and epidural abscesses cause significant morbidity and mortality among people who inject drugs (PWID). SIRI hospitalizations have risen and are opportunities to engage people in substance use treatment alongside infectious disease care. Treatment of SIRIs typically requires 2 to 6 weeks of antibiotics, often administered intravenously in the hospital, skilled nursing facilities, or at home. Increasingly, some SIRIs are treated with oral antibiotics [1, 2]. There is a growing focus on delivering medications for opioid use disorder (MOUD) alongside infectious disease treatment for patients with SIRIs [3]. Evidence suggests that MOUD decrease re-hospitalizations and mortality [4–6] following SIRIs. It is now standard of care to provide MOUD during hospitalizations for complications of substance use, including SIRIs [3]. However, these medications are underused; in Massachusetts, just half of those with SIRIs received MOUD in the year following hospitalization [7]. Addiction Consult Services address this gap by providing specialized, evidence-based hospital care and facilitating linkage to community-based treatment [8]. Some hospitals have even developed dedicated SIRI teams [2, 9]. However, there are opportunities to improve SIRI care. One study conducted at Boston Medical Center found that only 6 of 10 patients with SIRIs completed recommended antibiotic therapy despite the presence of an addiction consult service, and nearly 7 in 10 were re-hospitalized for injection-related infectious complications within 1 year [10].

Additional qualitative analyses are needed to explore how SIRI hospitalizations impact use patterns, harm reduction adoption, and views on MOUD [11]. Previous quantitative research on motivation during an SIRI hospitalization exists [12], but understanding participants’ perspectives through their own words is crucial to developing interventions that can improve post-hospitalization outcomes. Thus, we conducted a qualitative study of patients with recent SIRIs at Boston Medical Center, an urban safety-net hospital with robust addiction and infectious disease resources serving a diverse, publicly insured patient population. The objective of this study was to explore patient perspectives on substance use, MOUDs, and harm reduction following hospitalization for SIRI to inform future interventions.

## METHODS

### Study Design, Sample, and Data Collection

This qualitative study was designed to elicit the perspectives of post-hospitalization patients with recent SIRIs to assist in developing a longitudinal post-hospitalization support program. Boston Medical Center (BMC) has inpatient infectious diseases and addiction medicine consult services [13] that provide linkage to outpatient care [14]. The addiction consult service is comprised of physicians, nurse practitioners, social workers, and peer support specialists who work to initiate MOUD and provide harm reduction counseling.

Eligibility requirements included age 18–65, English or Spanish speaking, and having a qualifying SIRI such as endocarditis, bloodstream infection, osteomyelitis, epidural abscess, or septic arthritis related to injection drug use diagnosed at least 2 weeks but no longer than 3 years before recruitment. Potential participants from BMC were identified in several ways: (1) a medical record review using International Classification of Diseases, 10th Revision, codes for SIRIs and OUD between January 1, 2020, and January 1, 2023; (2) the infectious diseases outpatient parenteral antibiotics tracking list with evidence of OUD; (3) direct recruitment of individuals hospitalized with a qualifying SIRI and OUD; or (4) with self-reported qualifying SIRIs from Project TRUST, a drop-in harm reduction site. Recruitment was conducted via letter or telephone outreach or in person by trained study staff (A.M., A.R.).

All participants were administered a brief quantitative survey assessing demographics, substance use, and infectious disease histories (Appendix A). Data were collected in REDCap. Afterwards, study staff conducted interviews using a semistructured qualitative interview guide (Appendix B) focusing on the periods before, during, and after SIRI hospitalization and eliciting perspectives on their experiences and opportunities for improvement in care. The interview guide was developed by study staff with feedback from experts in infectious diseases and addiction medicine and was piloted and revised before use in the study. Interviews had a median duration of 38 minutes. Interviews were conducted via telephone or in person, and participants were compensated with a prepaid debit card of $40.00. The Boston Medical Center/Boston Medical Campus Institutional Review Board approved all study protocols.

### Data Analysis

Interviews were audio-recorded and professionally transcribed. Study staff verified transcriptions for accuracy, de-identified the transcriptions, and uploaded the files to a secure server for storage and analysis. Using an iterative, inductive process, study staff developed a codebook. Team members (S.D.K., A.M., A.R.) individually coded 3 interviews and met to compare resulting codes. Based on these codes, a preliminary codebook was developed and revised in consultation with a senior qualitative researcher (M.L.D.). Three interviews were initially recoded with the preliminary code book. Then, a fourth interview was coded to ensure consensus and finalization of the codebook. Using the finalized codebook, the remaining 26 interviews were individually coded using the qualitative coding software NVivo by the 3 members of the research team (A.R., G.V., A.M.) who met weekly with the principal investigator on the study (S.D.K.) to ensure consensus on coding. The study team then reviewed coded transcripts to identify key themes. This analysis focused on codes related to the impact of hospitalization on substance use, MOUD, and harm reduction perspectives. A member of the research team (T.J.) developed additional subcodes related to “the impact of hospitalization” to facilitate further thematic analyses while meeting weekly with the principal investigator (S.D.K.) until consensus. Once additional subcodes were finalized, members of the study team (T.J., S.D.K., M.L.D.) extracted core themes from the analytic sample.

## RESULTS

### Sample Characteristics

Among 30 participants, the median age was 39 years, and a majority were White Non-Hispanic (n = 21, 70.0%), straight (n = 23, 76.7%), cisgender males (n = 19, 63.3%), and English speaking only (n = 24, 80.0%). As seen in Table 1, most participants lived in their own house, apartment or condo, or in a house with others (n = 18, 60%); 12 participants (40%) were experiencing homelessness. All but 1 participant (n = 29) reported opioids as a preferred substance (97.7%), and 17 (56.7%) also reported a stimulant preference. The median number of lifetime SIRIs requiring medical treatment among the cohort (interquartile range) was 3 (2–10), including the qualifying hospitalization. Nearly all participants had been previously treated with methadone (n = 27, 90.0%) or buprenorphine (n = 26, 86.7%), and some with extended-release naltrexone (n = 8, 26.7%). At the time of the interview, 50.0% (n = 15) participants were treated with methadone, 26.7% (n = 8) with buprenorphine, 6.7% (n = 2) with extended-release naltrexone, and 16.7% (n = 5) were not treated with any MOUD. Most participants reported finishing their recommended antibiotic regimens for the qualifying infection (n = 24, 80%). The median duration from SIRI to interview (range) was 330 (14–1095) days.

**Table 1.** Characteristics of Participants With Recent Serious Injection-Related Infection (n = 30)

Characteristics	Interviewees (n = 30)
Median age, y	39 (26–58)
Type of infection, No. (%)	
Endocarditis	10 (33.33)
Bacteremia	5 (16.67)
Osteomyelitis	9 (30.0)
Epidural abscess	4 (13.33)
Septic arthritis	2 (6.67)
Source of recruitment, No. (%)	
CDW pull	12 (40.0)
BMC inpatient	8 (26.7)
OPAT	5 (16.7)
Community recruitment	4 (13.3)
Endocarditis list	1 (3.3)
Education, No. (%)	
Less than high school diploma	4 (13.3)
High school or GED	13 (43.3)
Technical/trade certificate	2 (6.7)
Some college or Associate’s degree	8 (26.7)
Bachelor's degree	3 (10)
Health insurance, No. (%)	
Medicaid	29 (96.7)
Medicare	1 (3.3)
Race + ethnicity, No. (%)	
White Non-Hispanic	21 (70.0)
Black or African American, non-Hispanic	2 (6.67)
Hispanic or Latino/a/x	4 (13.33)
Multiracial^a^	2 (6.67)
Other	1 (3.33)
Language, No. (%)	
English only	24 (80.0)
Bi/multilingual^a^	6 (20.0)
Living arrangements, No. (%)	
Living along in my own house, apartment, condo	13 (43.3)
Living in a household with other people	5 (16.7)
Residential facility with meals	2 (6.7)
Temporarily staying with a relative or friend	4 (13.3)
Temporarily staying in a shelter	5 (16.7)
Temporarily staying on the street	1 (3.3)
Employment status, No. (%)	
Full time	1 (3.3)
Part time	1 (3.3)
Not currently working	28 (93.3)
Receiving disability (SSI/SSDI), No. (%)	
Yes	9 (30.0)
No	21 (70.0)
Student, No. (%)	
Yes, part time	1 (3.3)
No	29 (96.7)
Gender, No. (%)	
Male	19 (63.3)
Female	9 (30.0)
Transgender male	1 (3.3)
Other	1 (3.3)
Sexuality, No. (%)	
Straight	23 (76.7)
Gay, lesbian, or homosexual	1 (3.3)
Bisexual	3 (10.0)
Others	3 (10.0)
Preference of substance, No. (%)	
Opioids only	12 (40.0)
Opioids + cocaine	15 (50.0)
Opioids + cocaine + methamphetamines/amphetamine	2 (6.67)
Methamphetamines	1 (3.33)
No. of infections, median [IQR]	3 [2–10]
	min: 1; max: 50
Type of antibiotics, No. (%)	
IV antibiotics	14 (46.7)
Mixture of IV and oral	16 (53.3)
Setting of antibiotics, No. (%)	
In the hospital	13 (43.3)
A mix of hospital, home, and a facility	17 (56.7)
Antibiotics finished, No. (%)	
Yes	24 (80)
No	5 (16.7)
Actively taking antibiotics	1 (3.3)
Have you ever been treated with methadone, No. (%)	
Yes	27 (90.0)
No	3 (10.0)
Longest time taking methadone, No. (%)	n = 27
<1 mo	2 (7.4)
1–6 mo	6 (22.2)
6 mo–1 y	2 (7.4)
1–5 y	11 (40.7)
≥5 y	6 (22.2)
Have you ever been treated with buprenorphine, No. (%)	
Yes	26 (86.7)
No	4 (13.3)
Longest time taking buprenorphine, No. (%)	n = 26
<1 mo	2 (7.7)
1 mo–6 mo	8 (30.8)
6 mo–1 y	0 (0.0)
1–5 y	11 (42.3)
≥5 y	5 (19.2)
Have you ever been treated extended-release naltrexone, No. (%)	
Yes	8 (26.7)
No	22 (73.3)
Longest time taking extended-release naltrexone, No. (%)	n = 8
<1 mo	3 (37.5)
1–6 mo	3 (37.5)
6 mo–1 y	2 (25.0)
1–5 y	0 (0.0)
≥5 y	0 (0.0)
Currently taking MOUD, No. (%)	
Methadone	15 (50.0)
Buprenorphine	9 (30.0)
Extended-release naltrexone	1 (3.3)
None	5 (16.7)
Preferred MOUD, No. (%)	
Methadone	17 (56.7)
Buprenorphine	8 (26.7)
Extended-release naltrexone	2 (6.7)
None	1 (3.3)
I don’t know/no preference	2 (6.7)
Outreach recruitment methods	
Clinical Data Warehouse	10 (33.3)
Outpatient parenteral antimicrobial therapy	9 (30.0)
Inpatient	6 (20.0)
Passive recruitment	5 (16.7)
Duration from SIRI to interview, median [IQR], d	330 [36–1082]

Abbreviations: BMC, Boston Medical Center; CDW, Clinical Data Warehouse; IQR, interquartile range; MOUD, medications for opioid use disorder; OPAT, outpatient parenteral antimicrobial therapy; SIRI, serious injection-related infection; SSI/SSDI, Supplemental Security Income/Social Security Disability Insurance.

^a^Eligible participants were recruited through telephone or letter via the Clinical Data Warehouse, the Outpatient Parenteral Antibiotic Treatment list, and the Endocarditis Working Group List.

**Figure 1.**
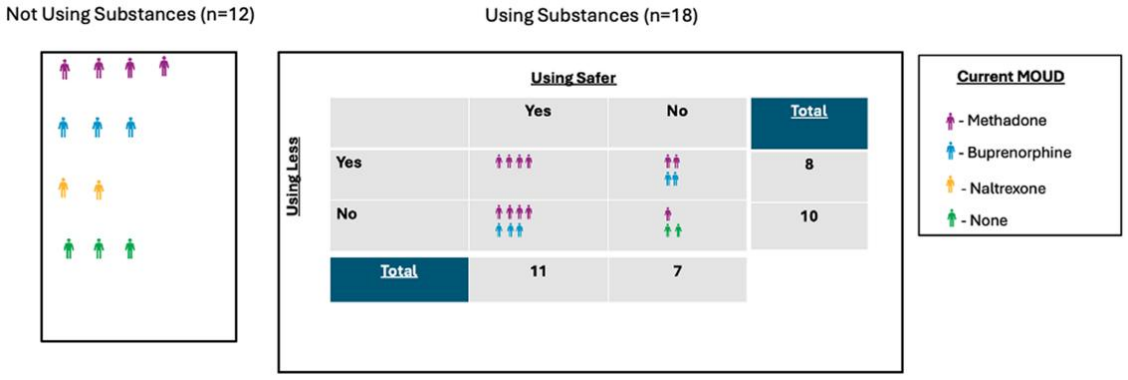
Substance use and harm reduction patterns following SIRI hospitalization as stated by participant (n = 30). Abbreviation: SIRI, serious injection-related infection.

During semistructured interviews, some participants said they stopped using after their hospitalization, while most participants said they continued to use. Of those who continued to use, a majority adopted harm reduction practices, several reported using less, and few said they did not use less or adopt safer practices (Figure 1).

### Qualitative Findings

Three key themes emerged from this analysis: First, there was a continuum of harm reduction practices post-hospitalization; second, participants had diverse perspectives on MOUD post-hospitalization; third, hospitalization can be an opportunity for reflection.

#### Theme 1. Harm Reduction Practices Were on a Continuum After SIRI Hospitalization

Participants’ post-hospitalization harm reduction practices existed on a continuum of using safer or not using safer (Figure 1). When asked about awareness of injection-related harm reduction practices and the usefulness of providing more harm reduction education following SIRIs, perspectives varied. Some participants said that they were unaware of certain harm reduction practices: “If I would have known licking my needle would have caused [the infection], I would have never done it. It was just a bad habit I had because I like the taste of the heroin” (ID #113), said a White man in his 50s. Another White man in his 50s said that information about harm reduction was readily available but not always utilized:

There’s plenty of information out there…. When you’re trying to take that pain away, you don’t care. You just want to take that sickness away…. The information is out there. There’s plenty of information. Believe me, people know. I’ve been in many programs…. They’re telling everybody, they’re keeping up to date with everything with the Narcan, with how to use clean needles and clean cookers, clean cottons, all that. So, no, I think they do a great job informing people around here. (ID #109)

Several participants reflected that their SIRI hospitalization led to adoption of harm reduction practices post-hospitalization. A multiracial female in her 50s discussed how the hospitalization prompted her to be more attentive to skin and needle hygiene: “If I didn’t go in the hospital, I would probably have been paralyzed. So I stopped sharing needles and start[ed] getting clean needles and stop[ped] using the same needle over and over” (ID #104).

A White Hispanic transgender man in his 20s discussed how even though he knew about harm reduction practices, implementing them was a challenge:

After the whole blood infection…I have to, like, maybe go into a bathroom that’s probably clean or…you can’t use at the shelter. So it was like trying to figure out where to use. Obviously, that was a little bit cleaner than outside, but also not get caught or get in trouble. You know, using…their resources to get clean needles as well. (ID #124)

Participants frequently moderated their substance use and adopted harm reduction behaviors following an SIRI hospitalization, but harm reduction knowledge and feasibility of implementation varied.

#### Theme 2. Participants Had Diverse Perspectives on MOUD After Hospitalization

In this cohort with significant MOUD experience, participant perspectives on MOUD following hospitalization for SIRI varied considerably.

#### Favorable Views of MOUD

Some participants felt that MOUD were extremely effective treatments. As a White female in her 30s explained, “I think [methadone] is a life-saving medicine…. I would’ve been 1000% dead right now if it wasn’t for methadone” (ID #123). Participants often discussed the impact of MOUD on improving daily functioning: One White male in his 30s stated, “I think methadone saved my life…. I’m actually able to go through the day and feel normal and not be sick…. It’s a miracle drug” (ID #126). Others even challenged the stigma of MOUD. As a White woman in her 50s explained:

I think they’re great. I think that you are still sober if you are taking a replacement therapy, like Vivitrol, Suboxone, methadone. I think they have saved many lives, and I totally disagree with people who claim you’re not sober if you take methadone or Suboxone or whatever. (ID #128)

#### Unpleasant but Necessary

Others recognized the health benefits of MOUD but acknowledged the challenging treatment structure. A White man in his 40s discussed how he found receiving treatment at the methadone clinic unpleasant and restrictive, but necessary to control withdrawal and maintain stability: “It’s a lot of work. You have to go to the methadone clinic every day. Sometimes you go wait in line for an hour. But it keeps me from being sick and making rash decisions” (ID #143). A White female in her 20s also commented on the inflexible nature of methadone treatment, discussing the concept of “liquid handcuffs”:

I hate having to go. It’s like you’re chained to it, pretty much like what they say its liquid chain[s]. You have to go every day, and you can [get] take home[s] but it takes a while…. But other than that, I have found methadone to be the most effective for me. (ID: #106)

Many participants expressed mixed perspectives on MOUD. Although they recognized functional benefits, perceived dependence on MOUD made them feel like they were swapping one drug for another. A White Hispanic transgender man in their 20s explained:

I’m conflicted because I still feel like I’m substituting, like, one that’s made in the lab for something I can buy off the street…. I just feel like, I’m swapping my fentanyl habit for…a Suboxone habit. But this has always been my problem with…opiate medicines…. I have friends that go to the methadone clinic, and they’re actually addicted to the methadone, not even the dope…. I just don’t wanna be like that. (ID #124)

Despite injection drug use complications and receiving care in a setting with robust MOUD resources, ambivalent perspectives on MOUD persisted in people post–SIRI hospitalizations.

#### Theme 3. Hospitalizations Can Be an Opportunity for Reflection

Most participants discussed how the hospitalization for their SIRI led to significant reconsideration of their substance use and overall health. A new environment and interruption in typical use patterns brought on by the hospitalization provided an opportunity for reflection. Some identified that a near-death experience during their hospitalization served as a particularly impactful warning.

#### Hospitalization as a Stable Environment

This cohort frequently discussed how homelessness contributed to their substance use. As a Hispanic male in his 30s explained, “It was really hard because I wanted to be clean…. In the place where I sleep and live is in the shelter, they had drugs in there, so it was really hard for me” (ID #133). Participants noted that the hospitalization provided relief from this instability, allowing them to reflect on their substance use and overall health. A Black man in his 50s described his circumstances similarly:

I think every time I go in there, it gives me time to reflect and to realize what and how I’m going…. You know, it’s like being in a dirty room and you’re depressed. If you clean up the room and if you take a shower and you put on some clean clothes, you have things around you, you start to feel better and you start to look at things in a different perspective. (ID #103)

#### Insight Into Severity of Condition

For some, reflection during the hospitalization provided insight into the severity of their illness and declining health. A Hispanic male in his 30s explained:

I feel like a light switched on in my head where the real seriousness of my disease and my infection really hit home…where I had to decide if I really wanted to live or if I wanted to continue living the way I was living, which wasn’t really living…was just existing. So the light bulb went off, and I decided to actually get my life together. (ID #134)

A third of individuals (n = 10) in this study had endocarditis, a heart condition that participants understood to be particularly dangerous. One White female in her 20s explained: “They told me if I had got endocarditis again, that, you know, I probably won’t make it…. I [will need to] have heart valve replacement surgery soon” (ID #106). Discussion of a near-death experience motivating participants was common. As another White female in her 20s explained:

It sent me back to reality. It scared me…. I don’t have any desire to use. I don’t want to use. I have not used since I left the hospital…. I don’t want to die…. It scared the crap out of me. It’s the first serious thing I’ve ever gone through. It’s the first time I’ve literally almost died. (ID #118)

#### A Break From Using and a Reachable Moment

Participants frequently discussed how the hospitalization interrupted their substance use patterns. A White female in her 30s described the hospitalization as an opportunity: “It was…good because it gave me…time away from using, and I was able to think clearly and not be fazed by…all the effects of everything” (ID #106). Meanwhile, others viewed the period of sobriety in the hospital as contributing to their goals of reducing or stopping use: “Because the first time I was in the hospital, I was there for like 2 and a half months. So it just proved to me that like, shit, I can go 2 months without using and be fine” (ID #123), explained a White female in her 30s. One White female participant in her 30s who reported using throughout her hospitalization still saw the hospitalization as a moment for linkage to treatment: “I wasn’t sober in the hospital. I used almost every day, but they talked me into going to treatment” (ID #120).

## DISCUSSION

In this qualitative study of people with recent SIRIs and high MOUD treatment rates, participants reflected back on their hospitalizations as opportunities to make changes to their health. While many reported stopping substance use post-hospitalization, most continued to use, sometimes using less or adopting new harm reduction practices. These findings suggest that hospitalizations for SIRIs can be an opportunity for patients to make changes in their drug use patterns and be a “reachable moment” [12] to foster patient reflection and education. Nonetheless, despite the opportunity to engage patients with SIRIs, ambivalence about long-term MOUD treatment and variability in harm reduction knowledge and implementation suggest potential areas of improvement in the crucial SIRI post-hospitalization phase.

Following hospitalization for SIRIs, many participants stopped using substances, and among those who continued to use, most reported using less or adopting harm reduction approaches. These findings, in addition to persistent MOUD stigma, provide further context to quantitative data surrounding MOUD treatment adherence. Most participants were not only motivated to change but reported that they implemented changes post-hospitalization. Some participants felt that information about harm reduction was novel, while others felt it was readily available. However, harm reduction awareness alone was not enough, as participants frequently discussed challenges in implementing these practices. This suggests a need for structural interventions to support the safety of individuals with recent SIRIs [15–17]. Interactions with hospital providers may be beneficial in educating patients about risks and safety in addition to linking to MOUD [18]. Documenting changes in substance use and harm reduction after SIRIs may help broaden definitions of success for clinicians and perhaps mitigate provider burnout [19]. This demonstrated ability to change behavior post-hospitalization is juxtaposed with this cohort’s ambivalence about MOUD in post-hospitalization discussion.

Most participants in this study were treated with MOUD, yet ambivalence about the treatment was common. MOUD initiation and linkage in the hospital did not emerge as key themes in this study. This could reflect high rates of MOUD treatment before the hospitalization or our focus on the impact of hospitalization rather than the hospital experience itself. Some participants held favorable views about MOUD, and others felt that MOUD was swapping one drug for another. Most viewed stopping MOUD as a goal for their long-term recovery. These perspectives likely reflect both negative personal experiences and stigmatized views concerning substance use and MOUD itself [20]. These views are common in other cohorts of people who use drugs and in certain treatment programs, where beliefs about MOUD use as incompatible with “abstinence” persist [21]. These findings suggest that an SIRI hospitalization, which carries a high mortality [22] and rehospitalization risk, may not alter these views [23]. Although regulation changes after the COVID-19 pandemic allowed increased flexibility with take-home doses [24], the methadone delivery system needs further reformation to improve patient experiences and accessibility. Longitudinal support following the hospitalization may improve MOUD engagement.

Most participants felt that their hospitalization led to reflection about their substance use and overall health. These findings corroborate evidence from other studies of patients who reported motivation to make changes while hospitalized [25–27]. Participants’ characterizations of their hospitalization as a temporary stable environment and catalyst for insight into their condition are a counterpoint to previous studies describing the hospital as a “risk environment” or a place that perpetuates stigma [28–30]. We present these findings of disproportionate positive experiences cautiously, as coding for this analysis focused mainly on post-hospitalization reflection and less so on the hospital experience itself. These findings may reflect improvements in hospital systems of care, selection bias related to study participation, or the hospital providing temporary shelter in a cohort experiencing homelessness. As this study was focused on the post-hospitalization experience, further exploration into the hospital experience itself is warranted.

This study has limitations. First, these participants were recruited in Boston, a city with relatively robust access to addiction treatment and harm reduction services that may not be generalizable to other locations. Although we recruited from multiple locations to represent diverse substance use experiences (eg, actively using, in recovery), those who participated may have had more resources (phone, harm reduction, etc). Second, despite robust training of interviewers, social desirability bias may have impacted participants’ responses. Third, though we used a collaborative approach to developing the interview guide and codebook, the clinical and personal experiences of researchers may have introduced bias into the structure of the guide or development of the codebook. As this study did not collect information on duration of antimicrobial therapy, future studies should explore the relationship between patient experiences and antibiotic treatment plans as oral antibiotics are more widely adopted for SIRIs [31, 32].

## CONCLUSIONS

Post-hospitalization participants with recent SIRIs looked back on their hospitalizations as a time for reflection that can lead to changes in substance use and harm reduction practices. However, ambivalence about MOUD was common, reflecting both stigma and frustrations with the treatment system. Hospitalizations for SIRIs are a reachable moment, but efforts to improve systems and provide longitudinal support are needed to promote harm reduction and MOUD retention.

## APPENDIX A

### Background

1. What is your age? _______
2. What is your highest level of education?
  - Less than high school diploma
  - High school or GED
  - Technical/trade certificate
  - Some college or Associate's degree
  - Bachelor's degree
  - Graduate/professional degree
3. What kind of health care insurance do you have?
  - Medicaid (eg, Mass Health, BMC Health Safety Net)
  - Medicare
  - Commercial health insurance (eg, Blue Cross, Tufts, etc.)
  - None
  - Don’t know
4. How would you describe your racial or ethnic identity? (you can choose more than one)
  - American Indian or Alaska Native
    - What is your tribal affiliation? ____________________
  - Asian
  - Black or African American
  - Native Hawaiian or other Pacific Islander
  - White
  - Hispanic or Latino/a/x
  - Other (specify ___________________________)
5. What languages do you speak (select all that apply)?
  - English
  - Spanish
  - Specify languages: ______________________________________
6. Which one of the following best describes your living arrangement?
  - Living alone in my own home, apartment, or condo
  - Living in a household with other people
  - Living in a residential facility where meals and household help are routinely provided by paid staff
  - Temporarily staying with a relative or friend
  - Temporarily staying in a shelter
  - Temporarily staying on the street
  - Other
7. Are you currently working?
  - Yes, full time (35 hours or more a week)
  - Yes, part time (less than 35 hours a week)
  - No
8. Are you receiving disability (SSI or SSDI)?
  - Yes
  - No
9. Are you a student?
  - Yes, full time
  - Yes, part time
  - No
  - Other
10. What is your current gender identity?
  - Male
  - Female
  - Transgender (please check this box in addition to any others)
  - Nonbinary or genderqueer
  - I am questioning/not sure of my gender identity
  - Other (specify ___________________________)
11. Do you consider yourself to be…
  - Straight or heterosexual
  - Gay, lesbian, or homosexual
  - Bisexual
  - Something else (specify)
12. Which substance to you prefer to inject?
  - Opioids
  - Benzodiazepines
  - Methamphetamines/amphetamines
  - Cocaine
  - Other
13. In your lifetime, how many times have you received medical treatment (including medications) for serious infections from injecting? _______ (specific to ed/hospitalization, make ordinal variables)
14. For your most recent infection, what kind of antibiotics did you receive?
  - IV antibiotics
  - Oral antibiotics
  - Mixture of IV and oral antibiotics
  - Other
  - None
15. Where did you receive these antibiotics?
  - In the hospital
  - At home
  - In a facility
  - A mixture of the hospital, home, and in a facility
  - Other
16. Did you finish the antibiotics that were recommended for you?
  - Yes
  - No
17. Have you ever been prescribed methadone?
  - Yes
  - No

17a. [If YES to q17] What is the longest time you consistently took methadone?
  - Less than 1 month
  - 1 month to 6 months
  - 6 months to 1 year
  - 1 to 5 years
  - 5 or more years

18. Have you ever been prescribed Buprenorphine (Suboxone, Sublocade)?
  - Yes
  - No

18a. [If YES to q18] What is the longest time you consistently took Buprenorphine?
  - Less than 1 month
  - 1 month to 6 months
  - 6 months to 1 year
  - 1 to 5 years
  - 5 or more years

19. Have you ever been prescribed Vivitrol?
  - Yes
  - No

19a. [If YES to q19] What is the longest time you consistently took Vivitrol?
  - Less than 1 month
  - 1 month to 6 months
  - 6 months to 1 year
  - 1 to 5 years
  - 5 or more years

20. Are you currently taking methadone, buprenorphine (Suboxone, Sublocade), or Vivitrol?
  - Yes
  - No

21. Which medication do you prefer to use?
  - Methadone
  - Buprenorphine
  - Naltrexone
  - None
  - I don’t know/no preference

## APPENDIX B

### RETAIN Interview Guide

Thank you for participating in our study. We are doing interviews to understand more about your experiences with opioid use disorder treatment during and after the infection you had from injecting drugs. We are talking to several people to learn about their experiences, so that we can understand how to support people better during these infections. Your responses and feedback today will be used to help improve treatment for others who have gone through what you have.

We are interested in your honest opinions and experiences, whether positive or negative. There are no right or wrong answers. We may share your responses to our questions in research articles. When we share your responses outside of our research team, your comments will be de-identified so that no one will be able to tell who said what.

Some of the questions I ask may deal with sensitive or private topics. You do not have to answer any questions that make you feel uncomfortable, and you can also stop or end the interview at any time. Please let me know if there is anything you don’t want to discuss or if you need to take a break.

I would like to record this interview. We will destroy the recordings after they are transcribed into text. To protect privacy, please try not to use identifying information like names. If you do, we will take them out when we review these conversations.

This interview will go over 3 topics, including your hospitalization, post-hospitalization, and your suggestions to improve care for these conditions. We have a list of questions to go through, and we may have additional questions based on what comes up.

What questions do you have before we start?

### Drug Use and Hospitalization

The first section is about your hospitalization and drug use.

1. Tell me about yourself. (Probe: anything else you want to share not covered in the survey; goal to facilitate discussion and put participant at ease)
2. Tell me about your drug use and how it was related to your hospitalization.
  - Why do you think you developed an infection this time and not other times you were using? (Probes: skin cleaning, missed shot, syringe not sterile, drug supply)
  - What drugs do you use most often? How do you use them? How long have you been using?
  - Why do you use drugs? How have they helped you or caused problems in your life?
3. Can you say more about why others may get infections like these?
4. What was it like for you in the hospital when you were treated for your infection?
  - How did you experience withdrawal and pain? Were your withdrawal and pain controlled? What were you treated with? How were you treated by staff?
  - How long do you think you had an infection before you came in? Why did you come in when you did? Why not earlier or later?
  - Did you think about using drugs while you were in the hospital? Did you bring drugs with you into the hospital or use other substances while you were admitted to the hospital?
  - Do you smoke cigarettes or vape?
    - If yes: How did you manage nicotine cravings while you were in the hospital?
5. How did health care providers talk to you about medications for opioid use disorder, like buprenorphine, methadone, or naltrexone?
6. How do you feel about using medications for treatment of opioid use disorder? (Probe based on responses in survey)
  - What is your experience with methadone?
    - If has experience: Can you tell me more about your experience? Were those experiences positive or negative?
  - What is your experience with buprenorphine, which is sometimes called Suboxone or Sublocade?
    - If has experience: Can you tell me more about your experience? Were those experiences positive or negative?
  - What is your experience with naltrexone, which is sometimes called Vivitrol?
    - If has experience: Can you tell me more about your experience? Were those experiences positive or negative?
7. How do you feel like your opioid use was addressed while you were in the hospital?
  - Was there someone you turned to or came to trust to discuss next steps with your substance use treatment? (Probes: family, friend, clinician, peer)
8. You needed long-term treatment with antibiotics. How did that go?
  - Was there someone you turned to or came to trust to discuss next steps with your antibiotics? (Probes: family, friend, clinician, peer)
  - How did the antibiotic treatment go? (Probe: if they finished, why/why not)
9. What happened to you when you were ready to leave the hospital?
  - How was the decision made about when you were ready to leave the hospital? Did the doctors tell you that you were ready to leave, or did you decide?
  - Where did you go when you left? Home, to a facility, some other place? How was that transition?
    - How did you feel about going there?

### Post-hospitalization and substance use treatment

Next, I’d like to talk about your post-hospitalization.

1. What was the plan for your withdrawal and pain management when you left the hospital?
  - What happened to your substance use treatment during that transition? What about your pain control?
    - What were you told would happen?
  - Tell me about your experience with that treatment.
    - Were you given options? Did you receive the treatment you wanted?
    - Did you go back to a place you had been previously treated?
    - Did you receive any referrals to treatment programs?
    - How long did you get that treatment?
    - Why did you continue or stop that planned treatment? (Probes: distance, housing, withdrawal, cravings, stigma, etc.)
    - If you stopped treatment, was it sudden? If you were treated with medications, did you taper? Did you return to using? (Probe: If residential, why did you leave?)
    - If you were already taking MOUD before your hospitalization, did your dose change? Did the providers talk about switching your medication?
2. How did the time you spent in the hospital affect your drug use?
  - Are you using in a different way than before your infection? (Probes: skin cleaning, amount, route, location, alone/with others)
  - Have you had additional infections since you were hospitalized?
  - Have you had overdoses since your hospitalization?
  - Did the hospitalization change how you think about your overall health? (Probes: health concerns, worries, or priorities)
3. Did the treatment providers you saw for your infection also address treatment for opioid use? Similarly, did treatment providers you saw for your opioid use address your infection?

### Improving Treatment for Opioid Use After Serious Infections

Next I would like to ask you about how to improve the treatment of people who are hospitalized for serious infections from drug use.

1. What would have worked better for you in treating your substance use after a serious infection like the one you had?
  - What could the programs providing care for you have done differently? (Probes: easier to access, telemedicine, higher doses, etc.)
  - What could you have done differently?
  - If you wanted to continue treatment for your opioid use disorder or start treatment after you left the hospital, did you know you could do that?
  - Would more information about safe injection or overdose have helped you?
  - What about access to syringes or naloxone in the hospital or right after?
2. Is there additional support you wish you had? (Probes: a consistent doctor or outreach worker/peer, a place to go, transportation, a phone)
3. What do you think of having someone like a peer or case manager who would reach out to you to support you after the infection and help you get connected to or stay in substance use treatment?
  - How best for them to contact you? (Probes: text, email, phone, in-person)
  - When and where would you want to see them?
  - How often would you want to have contact with them?
  - What kind of person would you want for this role? What you like that person to know or understand about your situation?
4. **Conclusion:** Is there anything else you would like to add to our conversation today?

Thank you so much for your time and energy today. We really appreciate all of the information that you have shared with us.
